# Stimuli-Responsive Polymer Brushes for Flow Control through Nanopores

**DOI:** 10.3390/jfb3020239

**Published:** 2012-03-26

**Authors:** Shashishekar P. Adiga, Donald W. Brenner

**Affiliations:** 1 Kodak Research Laboratories, Eastman Kodak Company, Rochester, NY 14620, USA; 2 Department of Materials Science and Engineering, North Carolina State University, Raleigh, NC 27695, USA; Email: brenner@ncsu.edu

**Keywords:** smart membranes, signal responsive polymers, polymer brushes, computational modeling, flow control, nanofluidic valve, simulation

## Abstract

Responsive polymers attached to the inside of nano/micro-pores have attracted great interest owing to the prospect of designing flow-control devices and signal responsive delivery systems. An intriguing possibility involves functionalizing nanoporous materials with smart polymers to modulate biomolecular transport in response to pH, temperature, ionic concentration, light or electric field. These efforts open up avenues to develop smart medical devices that respond to specific physiological conditions. In this work, an overview of nanoporous materials functionalized with responsive polymers is given. Various examples of pH, temperature and solvent responsive polymers are discussed. A theoretical treatment that accounts for polymer conformational change in response to a stimulus and the associated flow-control effect is presented.

## 1. Introduction

**Figure 1:**
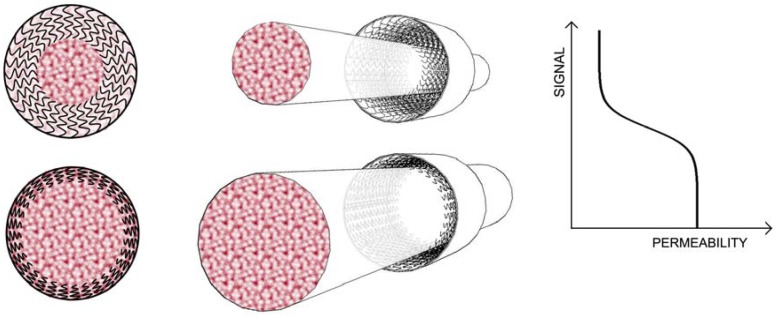
Schematic illustration of the smart flow control valve. The smart valve changes its permeability in response to external stimulus (pH, T, or Light) as the grafted chains in the interior of the pore undergo a stretched to collapsed transition and offer reduced resistance to flow.

Nanochannels that reversibly change their pore-size and/or permeability in response to environmental stimuli are an emerging class of smart materials with many potential applications [1,2,3,4,5,6]. One possible application is self-regulated drug delivery, in which transport of an encapsulated drug across a nanoporous membrane is regulated based on a change in a given physiologic parameter. The functionality of the majority of these smart nanopores are based on reversible expansion and collapse of responsive polymers incorporated into the membranes that regulate molecular transport through the pores and provide a chemomechanical effect (Figure 1). In addition to combining the sensor and actuator functionalities in one device in the smart drug delivery systems, there is a second intriguing application. These smart nanopores allow for programmable flow control in microfluidic chips where fluid/molecular transport can be turned on/off remotely, by providing an external stimulus [7].

These smart nanopores have been prepared by functionalizing the pores with a suitable responsive polymer that creates a switchable membrane permeability in response to variation in temperature [7,8,9,10,11], pH [12,13,14,15,16], ionic/solute concentration [12,17], and light [18,19]. Similarly, polymers that respond to electric fields [20,21] offer an additional triggering mechanism. In general, three distinct approaches have been used to functionalize membranes with smart polymers. In one widely used method, polymer chains are densely end-grafted onto the surface to form a polymer brush layer inside the pore and/or on the surface of the porous support. The brush layer can stretch and collapse in a reversible manner to open and close the pores in response to a stimulus. In the second method, responsive cross-linked polymer gels are incorporated into a nanoporous support. In the third method, porous membranes are modified by depositing layer-by-layer assembled polyelectrolyte multilayers inside pores; changes in film thickness are utilized to produce membrane responsiveness. In contrast to conventional network structures in the two latter techniques described above, which have relatively rigid chain ends, the end grafted polymer brushes exhibit rapid conformational changes [4] and hence a superior response time.

Many technological fields including drug delivery, chemical analysis, separation, and nanotechnology stand to benefit greatly from nanoscale signal-responsive flow control provided that precise control over molecular scale transport through nanopores is achieved. To this end, it is important to establish a fundamental understanding of conformational response of grafted polymers inside pores to external stimulus and how it affects fluid/molecular transport—the subject of this review article. We begin by providing a brief overview of polymer brushes and their responsive mechanisms. Using a model developed within a theoretical framework based on mean-field theory and flow through porous medium, we will examine the effect of an external stimulus quantified in terms of a very generic solvent quality parameter on the polymer brush conformation and how it alters the permeability of a channel. Through our analysis, we will demonstrate that a small change in the solvent quality, brought about by a change in temperature, pH or salt concentration can cause a significant permeability change. Our theoretical analysis shows that by judicious choice of the responsive polymer/solvent system and the grafting parameters, it is possible to design a smart nanoporous system to respond to cause permeability change to a specific extent.

## 2. Polymer Brushes and Their Responsive Mechanisms

Central to the fabrication of smart nanovalves is functionalizing the pore surfaces with responsive polymer brushes. Polymer brushes are formed by end-grafting polymer chains to a surface at high densities such that the average distance between two grafting points is smaller than the average equilibrium size of an isolated chain [22,23,24]. In a good solvent, the polymer chains in the brush layer are forced to stretch away from the grafting surface to minimize excluded volume interactions. Polymer brushes are formed by either grafting to or grafting from techniques. In the former approach, preformed chains are end attached to the grafting surface via physisorption or chemisorption. While this method offers a very simple method to form brush layers, as highlighted in a recent review by Barbey *et al*. [25], the grafting density that can be achieved is limited due to steric repulsion experienced by new chains due to already grafted chains. In the latter approach, the polymerization is initiated on the initiator-functionalized surface. This approach offers superior control over layer thickness and density.

The conformation of polymer chains is determined by their solvent environment and for a specific polymer-solvent system the relative strengths of interactions between polymer segments *vs*. polymer-solvent dictates the overall chain conformation. These interactions can be steric, van der Waal’s or electrostatic in nature. If we considered steric interactions, which arise from the fact that polymer segments have a finite volume and exclude other segments from occupying this volume, they tend to expand polymer chains from their ideal chain configurations. For this reason, steric effects are also termed excluded volume effects. In the presence of a good solvent, the polymer chains maximize the solvent contacts and swell, while in a poor solvent the chain collapses to reduce polymer/solvent interactions. The overall conformational change in the polymer relies upon the signal responsiveness of the functional groups, for example, acidic or basic groups that respond to pH, groups interacting via hydrogen bonding or van der Waals forces with the solvent that respond to temperature, or photolabile groups that respond to light [26,27,28,29]. 

In the case of thermo-responsive polymers, a stretch to collapse transition occurs at a transition temperature due to a change in the solvation state. Polymers with a lower critical solution temperature (LCST) undergo a collapse transition when the temperature is increased above the transition temperature. The LCST behavior arises due to specific interactions such as the hydrogen bonding between the polymer and the solvent that become unfavorable above the critical temperature. For example, the water soluble poly(N-isopropylacrylamide) (PNIPAAm) has a LCST around 32 °C above which it becomes immiscible in water [30]. The phase transition occurs as the gain in enthalpy due to hydrogen bonds between the polymer and water molecules is overcompensated by gain in entropy by breaking them. Polymers that become soluble upon heating have an upper critical solution temperature (UCST) [31]. A typical UCST system is a zwitterionic polymer, in which electrostatic interactions that stabilize the collapsed state at temperatures below the transition temperature become unfavorable above that temperature. Polymers with dispersive interactions with the solvent can also exhibit a UCST behavior.

**Figure 2:**
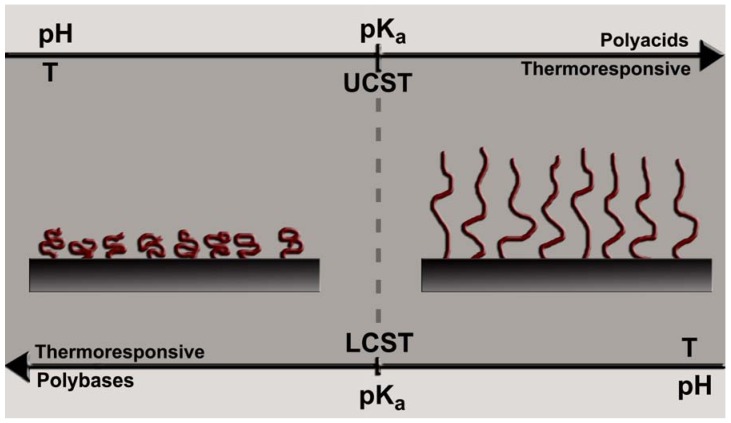
Thermo and pH responsive behavior of polymers. The polymers that exhibit LCST behavior undergo a collapse transition when heated above the critical temperature. The polymers with UCST undergo a collapse-stretch transition when heated above the critical temperature. In the case of pH-responsive polymers, a polyacid collapses when the pH is lowered to values below its pK_a_ value. On the other hand, a polybase collapses when the pH is increased above its pK_b_ value.

pH-responsive polymers are based on weak polyelectrolytes [27,28,31], which carry ionizable repeating units, and the number of groups ionized strongly depends on pH and ion concentration of the solvent. Polyanions such as poly acrylic acid and poly sulphonic acid are ionized in neutral and alkaline pH and electrostatic repulsion between charged segments result in their expanded conformations. As the pH is lowered below the pKa (acid dissociation constant) of these molecules, the ionized groups become protonated causing a collapse transition. In contrast, poly[2-(dimethylamino)ethyl methacrylate] (PDMAEMA) and poly(vinylamine) (PVAm) polymers, which display basic functional groups become ionized at low pH values and undergo a collapse transition due to deprotonation of cationic groups upon increasing the pH values. In addition to pH, polyelectrolytes are also sensitive to variation in ionic strength. The thermo and pH responsive behavior of polymer is summarized in Figure 2.

Light-responsive linear polymers possess photo-responsive units such as azobenzene [18], spiropyran [19]. On exposure to irradiation, reversible isomerization results in changes in conformation, viscosity and polarity.

### 2.1. Thermoresponsive Flow Control

Temperature-sensitive grafted polymers have been exploited to achieve flow regulation in synthetic membranes. A majority of previous studies involving thermoresponsive permeability control has exploited the LCST behavior of PNIPAAM [7,8,9,10,11]. For example, Akerman *et al*. [9] grafted PNIPAAM chains into poly(vinylidene fluoride) (PVDF) membranes and demonstrated that transport of model compounds across the grafted membranes can be controlled as a function of temperature. The flow regulation was shown to be a function of grafting density, degree of polymerization, and ionic concentration. In another study, Lokuge *et al*. [11] have achieved actively controlled thermoresponsive, size-selective transport control by grafting PNIPAAM brushes onto a Au-coated nanocapillary membrane through the use of atom transfer radical polymerization (ATRP). While the open-close transition of PNIPAAM functionalized membranes occurs at 32 °C which corresponds to its LCST, Xie *et al*. [32] have demonstrated that by adding varying amounts of hydrophilic or hydrophobic monomers into PNIPAAm it is possible to increase or decrease the transition temperature. The LCST was found to increase linearly with increasing molar percentage of the hydrophilic monomer and decrease linearly with increasing molar percentage of the hydrophobic monomer. Also, Rao *et al.* [33] fabricated functional membranes containing elastin-like polypeptides in the place of PNIPAAM. Elastin-like polypeptides may exhibit a wide range of lower critical solution temperature values, depending on the primary sequence and the length of these materials. Thus it is possible to design and fabricate a temperature-responsive gating membrane with the desired transition temperature specific to a given application. 

### 2.2. pH-Responsive Flow Control

pH-sensitive materials have also been utilized to achieve flow regulation in synthetic membranes. Ito *et al.* [12] have investigated pH-triggered water permeation control through membranes grafted with three different polyacids. They demonstrated the change in permeation occurred at pH values of 3.0, 4.0 and 6.8 for poly(acrylic acid), poly(methacrylic acid) and poly(ethacrylic acid) grafted membranes, respectively. Subsequently, they demonstrated a pH-responsive nanometer-scale gate, in which a polypeptide brush was grafted into a gold plated nanoporous membrane [15]. Water permeation through the material was regulated by helix-coil transformation of grafted poly-(L-glutamic acid) chains in response to pH. At low pH values, grafted poly-(L-glutamic acid) has a helical conformation. As the pH is increased, the helical conformation expands to a random coil conformation. This transition reduces the effective pore diameter and hence the system permeability. Mika *et al*. [14] synthesized smart membranes with inverse pH behavior. They reported that polypropylene microfiltration membranes containing poly(4-vinylpyridine) anchored within the pores exhibited very large chemical valve effects, specifically, an increase pressure-driven permeability by more than three orders of magnitude when the pH was increased from two to five [14]. Qu *et al*. [34] demonstrated a novel composite membrane system that utilized both grafted polymers and gels to produce a large pH-responsive release. Their membrane consisted of a porous polyvinylidene fluoride (PVDF) membrane grafted with positively pH-responsive poly(methacrylic acid) (PMAA) linear chains. These smart pores in the membrane acted as flow control valves to a reservoir inside which a crosslinked, negatively pH-responsive poly(N,N-dimethylaminoethyl methacrylate) (PDM) hydrogel functioned as a functional pumping element. The cooperative action of the pH-responsive gating and pumping systems produced responsive release rates much higher than either of the mechanisms used alone. Next generation nanoporous materials are envisioned with multiple biological functionalities, including size screening, responsive flow regulation, and dynamic pore sizing.

## 3. Theoretical Analysis

**Figure 3:**
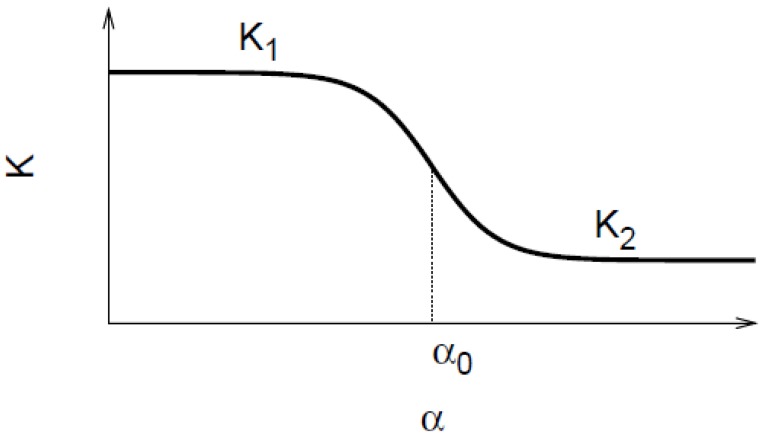
The sigmoid behavior of the smart nanovalve. The permeability change in response to a stimulus is depicted schematically.

To design smart nanovalves or responsive membranes, one must consider the following factors: (i) critical value of the stimulus at which the pore responds; (ii) the sharpness of the transformation; (iii) the permeability/flow rates in the open state as well as in the closed state—these determine the valve effect or the on/off ratio. The signal responsive permeability change through a nanopore is best described by a sigmoid curve (Figure 3) as follows,

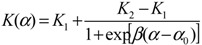
(1)

With reference to Figure 3, the parameters, *K*_1_, *K*_2_, represent the permeability values in the open and the closed states of the pores, respectively. The parameters *α*_0_, and *β* represent the inflection point and the sharpness of the permeability change, respectively. The sign of *β* of determines whether the change is positive or negative. A control over all these parameters is possible by the proper choice of the polymer/solvent system, the grafting density, chain length and the pore diameter. It is clear that understanding and control of transport on the nanoscale will enable the construction of novel devices for specific applications in sensing, delivery, separations and analysis. Thus, it is critical to develop modeling techniques to gain a fundamental understanding of nanoscale flow control that leads to the design of polymer grafted nanoporous systems for target applications. 

**Figure 4:**
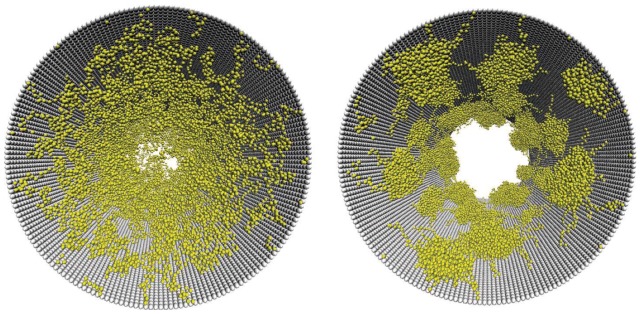
Coarse grained MD simulations of polymer grafted nanopores**.** The cross sectional snapshots of the nanopore depict conformations of grafted polymers in a good solvent (left) and in a poor solvent (right). The monomers (yellow) and wall atoms (grey) are shown. The solvent particles are omitted for clarity. Reprinted with permission from reference [36]. Copyright (2005) American Chemical Society.

**Figure 5:**
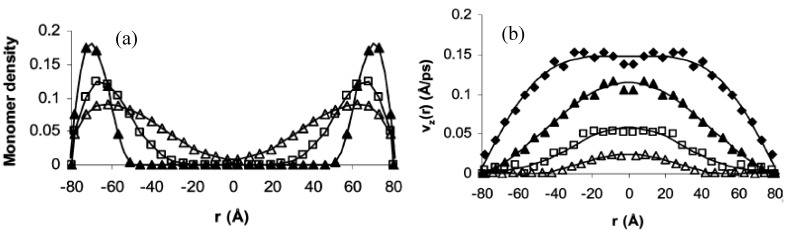
Monomer density and solvent flow profiles from coarse grained MD simulations**.** (**a**) Monomer density profiles for systems A (filled triangles), B (open squares) and C (open triangles). The polymer-solvent interaction strength increases from A through C; (**b**) The corresponding solvent velocity profiles across the pore for A (filled triangles), B (open squares) and C (open triangles). For comparison, velocity profile of an unmodified pore under the same flow field is shown (filled squares). Reprinted with permission from reference [36]. Copyright (2005) American Chemical Society.

Recent computer simulations have probed the role of grafted polymer coatings in modulating flow through nanochannels. For example, we have used coarse grained molecular dynamics (MD) to illustrate that grafted polymers offer resistance to flowing particles that changes according to their conformation providing smart flow control functionality to the nanoporous membrane [35,36]. Essentially, the grafted polymers extend from the interior of the nanopore to a degree determined by the strength of their interaction with the solvent (Figure 4). Thus, in a good solvent the monomer density profile extends farther towards the center of the pore as compared to a poor solvent, in which the polymers are collapsed that leaves the central region of the pore open (Figure 5a). The stretched and collapsed conformations lead to drastically different velocity profiles of a fluid flowing through the pore (Figure 5b). The monomers can be viewed as particles that exert hydrodynamic drag on the solvent. A good solvent experiences this drag across a larger cross section of the pore as compared to a poor solvent. The flow rate through the nanopore can be changed continuously and reversibly between the fully collapsed (open) and fully extended (closed) conformational states by tuning the polymer-solvent interaction strength.

In addition to our work, MD simulations have also been used to investigate modulation of electroosmotic flow through nanopores with grafted polymer chains [37]. Huang *et al*. used dissipative particle dynamics (DPD) simulations to model signal-responsive flow control in polymer grafted slit-pores [38]. While these detailed simulations have provided fundamental information on polymer-flow interaction at the molecular level, they are computationally expensive to apply to design specific polymer-solvent systems. To address this, we previously developed a continuum hydrodynamic method that incorporates environment-triggered change in conformation of the grafted molecules to calculate flow control through these smart pores [39]. Specifically, we modeled the pH-responsive flow control through a poly-L glutamic acid grafted cylindrical nanopore by taking into consideration the helix-coil transition of the grafted layer. 

### 3.1. Modeling Approach

**Figure 6:**
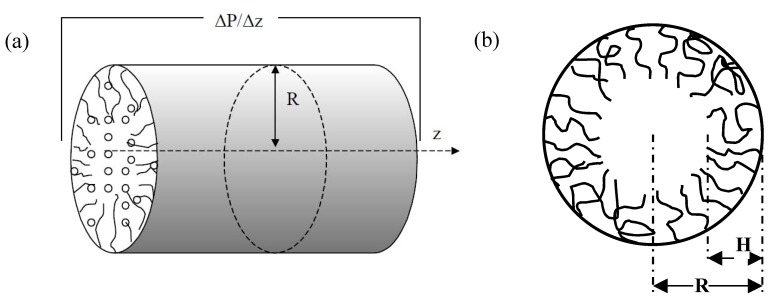
A schematic representation of the polymer grafted nanopore. The permeability change in response to a stimulus is depicted schematically.

Our model considers flow through a cylindrical nanopore grafted with polymer chains (Figure 6a). Following earlier continuum calculations [40], this problem is treated as flow through a porous medium and the grafted polymer chains are approximated as a polymer brush layer with permeability related to the monomer volume fraction. The resulting Brinkman equation [41] for the system can be written as

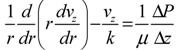
(2)
where *r* is the radial distance from the center of the pore, μ is the viscosity of the solvent, ∆P/∆z is the pressure drop driving the flow, *v_z_* is the z-component of solvent velocity, and *k* is the permeability of the brush layer. This continuum equation completely describes flow through a cylindrical nanopore grafted with a polymer brush by assigning an infinite *k* value for the central polymer-free region of the pore (which reduces equation (2) to the Navier-Stokes equation), and a finite value for permeability through the brush layer that is related to the monomer volume fraction *Φ* by some function *k*(*Φ*) to be described. It is important to point out that *Φ* is not a constant across the grafted layer thickness (step function profile), and in fact has a parabolic profile under good solvent conditions as predicted by self-consistent mean-field theories [24,42] and supported by neutron reflectivity measurements [43]. This implies that the permeability *k* through the grafted layer is a function of *r*. The monomer volume fraction profile *Φ*(r) in the brush layer is determined by extending a mean field analytic brush model [42] to polymer chains grafted to a concave surface. This model takes as input the pore size, polymer grafting density, number of monomers per chain, monomer repeat length, and Kuhn length. To incorporate the effect of solvent quality on *Φ*(r), the mean-field theory of polymer brushes is used. Equation (2) is then solved numerically to determine the velocity profile and hence the fluid flow rate through the pore as a function of pH.

### 3.2. Calculation of the Monomer Density Profile

All self-consistent mean field theories of polymer brushes involve expressing the free energy as a functional of the monomer volume fraction profile in the grafted layer and minimizing this functional under appropriate constraints to obtain the equilibrium volume fraction profile [24]. Here, we calculate *Φ*(r) by extending the mean field analytic theory of Zhulina and co-workers [42] developed for planar brushes to the case of a concave cylindrical brush. It is worthwhile to point out that a computationally more demanding numerical SCF approach based on Scheutjens and Fleer [44] can be employed to determine the structure of grafted polymers inside cylindrical pores by Egorov *et al*. [45]. We will determine the dependence of the equilibrium structure of the concave polymer brush on the grafting density, length of grafted chains, curvature of the cylinder, and solvent quality. In the present work, we will ignore the effect of flow field on the brush structure. While at relatively high Weissenberg numbers the flow field is known to cause shear induced deformation of the brush [36], it is reasonable to consider the equilibrium brush profile at low shear values.

We consider polymer chains of length *N* monomers grafted to an impermeable concave cylindrical surface of radius *R* (Figure 6a,b), with a grafting density of one chain per an area of *s*. The monomer size and the number of residues in the Kuhn length are taken as the effective monomer size *a* and thenumber of residues *p* in the effective Kuhn length respectively. The quantity *r* is the radial distance measured from the axis of the cylinder and *h* = *R* − *r* is the distance from the grafting surface. To determine the monomer density profile, *ϕ*(*h*), as a function of distance from the grafting surface we first define the total conformational free energy of the concave brush,

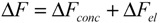
(3)
which contains the volume interaction energy,

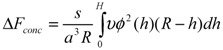
(4)
and elastic stretching energy

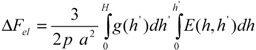
(5)
where the function g(h^’^) is the average fraction of chains ending at a radial distance h^’^ and E(h, h^’^) gives the local stretching dh/dn at h for a chain ending at h^’^. The parameter υ = *πa*^3^/6 represents the excluded volume and *H* is the distance from the grafting surface beyond which the density of monomers is zero (Figure 4b). The volume fraction of monomers *ϕ*(*h*) is expressed by the functions *g*(*h*^’^) and *E*(*h*, *h*^’^) as

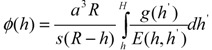
(6)

Minimizing the free energy Δ*F* as a functional of unknown functions *g*(*h*^’^) and *E*(*h*, *h*^’^) under the constraints

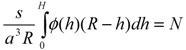
(7)
and

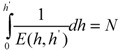
(8)
gives the expression for the function of local chain stretching

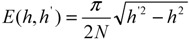
(9)
and for volume fraction of monomers in a layer

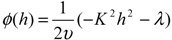
(10)
where

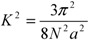
(11)
and *λ* is a undetermined Lagrangian multiplier that can be determined from the normalization Equation (7). The function *g*(*h*^’^) is determined by solving the integral Equation (6), with *g*(*h*^’^) as an unknown function. Equations (9), (4) and (5) were then used to determine the free energy Δ*F* as a function of height *H*. The equilibrium value of brush height, *H*_0_ was determined by minimizing the free energy with respect to *H*. Once *H*_0_ is known, the monomer volume fraction profile is easily computed.

**Figure 7:**
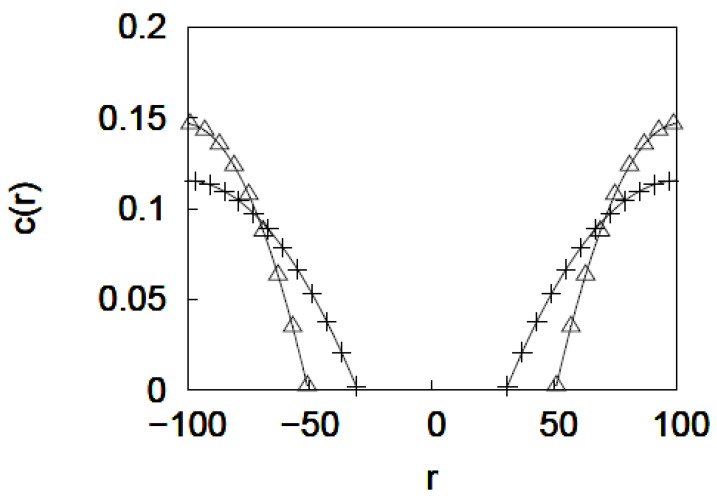
Monomer density profile for a polymer brush grafted inside a cylindrical nanopore of radius R = 100 *a* as a function of solvent quality, ν = 1 (open triangles), and 0.4 (plus signs). r is the distance from the center of the pore.

As an illustration, we have calculated the monomer density profile for a polymer brush grafted inside a cylindrical nanopore of radius R = 100 *a* for solvent quality parameter values of ν = 1.0 and 0.4. The values N = 400 and a grafting density = 0.01 *a*^−2^ were used. The monomer density *c*(*r*) = *ϕ*(*r*)/*πa*^3^/6 plotted in Figure 7 for different values of ν shows spreading of the grafted layer as the solvent quality is decreased. This indicates that for the brush considered in the calculation increasing ν decreases the pore opening. It is important to note that unlike the monomer density profile obtained from MD simulations (Figure 4a), which increases from zero at the grafting surfaces to reach a maximum value before decreasing, the density profile from mean field calculations is maximum at the grafting surface and then decreases parabolically. This discrepancy arises due to the fact that the mean-field theory calculation does not take into account the interaction between the grafting surface (pore interior) and the monomers. It is possible to include such an interaction in numerical self-consistent field calculations [45]. 

### 3.3. Calculation of the Velocity Profile

**Figure 8:**
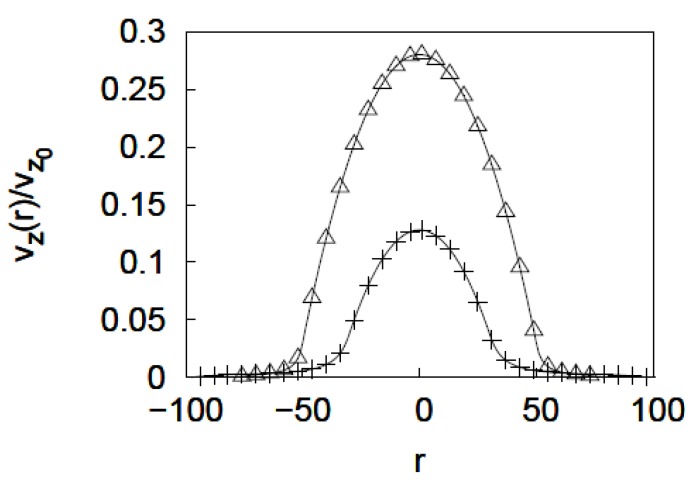
Normalized velocity profiles calculated using the Brinkman equation. *v*_z0_ is the velocity at the center of the pore in an unmodified pore. The velocity profiles are for ν = 0.4 (open triangles) and 1 (plus signs) for a pore of R = 100 *a*, *N* = 400, and grafting density = 0.01 *a*^−2^.

Calculation of the velocity profiles used as an input *Ф*(*r*) obtained from the mean-field method was explained in the preceding section. Due to the variation of polymer volume fraction as a function of *r*, Equation (2) must be solved numerically. The calculation was performed by dividing the brush region into *n* annular shells of thickness *dr*. A value of *dr* = *a* was used. The Brinkman equation was written for *i* = 1, *n* shells separately with permeability value *k*_i_ related to the monomer density of the corresponding shell as *k*_i_ = *a*^2^/*Ф_i_^2^* [39]. For shells free of polymers, equation (2) reduces to the Navier-Stokes equation. The solution for the velocity profile involved making an initial guess for the velocity at the center of the pore. Equation (1) was then applied to shells *i* = 1 (*r* = 0) to *n* (*r* = *R*) sequentially, subject to boundary conditions of constant velocity and shear stress at the interface between shell *i*-1 and *i* to obtain the velocity profile inside the pore. The composite velocity profile across the nanopore was obtained by successively improving the guess until the boundary condition that velocity at *r* = *R* vanishes was met. As an example, the velocity profiles at ν values 1 and 0.4 for a pore of R = 100 *a*, grafting density = 0.01 *a*^−2^ and degree of polymerization *N* = 400 are plotted in Figure 8. The velocity profile is normalized by v_z0_, the velocity at the center of an unmodified pore under the same flowing conditions. When the grafted layer is expanded it reduces the flow region and hence, also, reduces the fluid velocity at the center as compared to the collapsed brush layer. The overall effect is that as the solvent quality improves the flow rate through the nanopore decreases.

**Figure 9:**
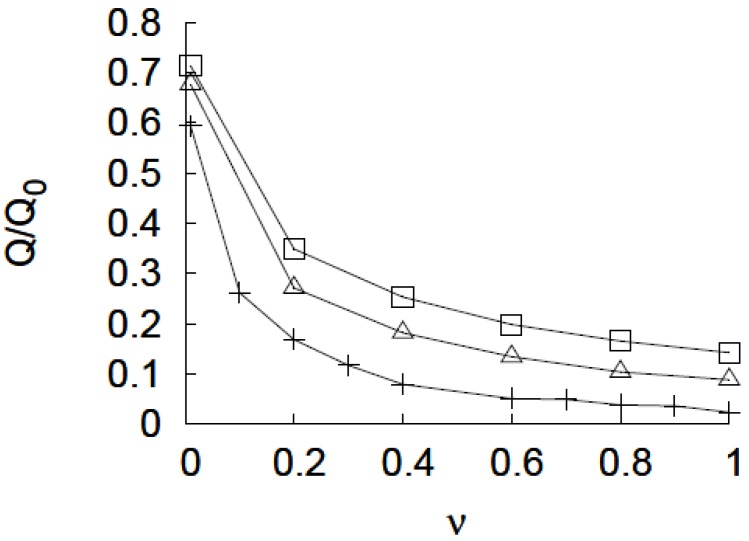
The variation of normalized flow rate with solvent quality. The flow rate is normalized with respect to the flow rate through an unmodified pore. The pore radius R = 100 *a*, *N* = 400, and grafting density = 0.01 *a*^−2^ (plus sign), 0.005 *a*^−2^ (open triangles), 0.0033 *a*^−2^ (open squares).

This is illustrated in Figure 9 where the normalized flow rate as a function of υ is plotted for R = 100 *a*, *N* = 400 and grafting density values of 0.01 *a*^−2^, 0.005 *a*^−2^ and 0.0033 *a*^−2^. The analysis also illustrates that a higher grafting density leads to a lower open pore permeability as well as a higher on/off ratio. A similar analysis of the permeability change with solvent quality can be performed for varying chain lengths. In principle, the smart nanopore can be designed to have any desired ratio of flow rate in open and closed configuration by choosing an appropriate pore radius and polymer content *P*_c_ (the amount of grafted polymer per unit pore volume = 2*N*/*sR*).

### 3.4. pH-Responsive Flow Gating via the Helix-Coil Transition of Poly(L-Glutamic Acid) Chains Grafted Inside Nanopores

**Figure 10:**
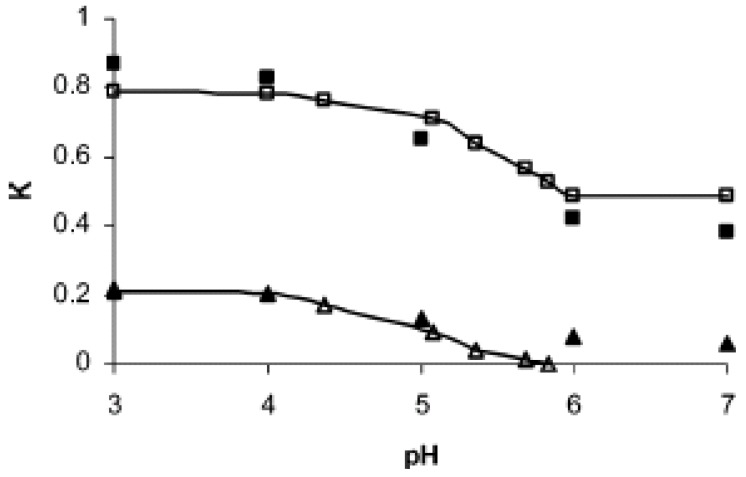
Flow control through the pH responsive poly(L-glutamic acid) grafted nanoporous system. The grafted PLGA chains undergo a helix to coil transition as the pH is increased. Comparison of pH dependent dimensionless permeability as predicted by the model and from experiments by Ito *et al*. [15] is given. Two systems with different grafting parameters investigated in their work are shown. The pore radius *R* = 800 Å. System A: *N* = 80, grafting density = 0.05 *a*^−2^; experimental (filled squares); model (open square). System B: *N* = 480, grafting density = 0.033 *a*^−2^; experimental (filled triangles); model (open triangles). Reprinted with permission from reference [39]. Copyright (2007) American Chemical Soceity.

The model described above can be applied to real flow control systems. The key is to incorporate the effect of signal responsive behavior of the structure of the brush layer. In the case of pH responsive systems, it is instructive to incorporate protonation/deprotonation as a function of pH and self consistently determine the monomer density profile in the polyelectrolyte layer. For example, recently Tagliazucchi *et al*. analyzed the pH-sensitive ionic conductivity through poly(4-vinyl pyridine) grafted nanopores. In particular, they determined the pH-sensitive conformation of the grafted brush by minimizing a free energy functional that incorporates interactions among the polymer, solvent, and the ions. Previously, we extended the model described in section 3.2 and 3.3 to analyze water permeation control through a nanoporous membrane grafted with poly(L-glutamic acid) chains [39]. We analyzed the helix-coil transition according to the Zimm-Bragg model to determine the monomer density profile of the grafted polypeptide layer inside a cylindrical nanopore as a function of pH. The flow rate through the nanopore was then calculated as a function of pH and the total permeability of the nanopore is plotted in Figure 10. These calculated pH-induced permeability change had a sigmoidal behavior and was in very good agreement with experimental results reported by Ito and coworkers [15]. The findings establish that polymer statistical mechanical models combined with a continuum porous layer treatment of flow through the polypeptide grafted nanopore can be used successfully to model smart flow control systems. The effect of grafting parameters on permeability change was analyzed. The optimum grafting parameters correspond to those for which the span of the monomer density profile is approximately equal to the pore radius in the closed state. The method described here can be easily extended to other polymer-solvent systems.

**Figure 11:**
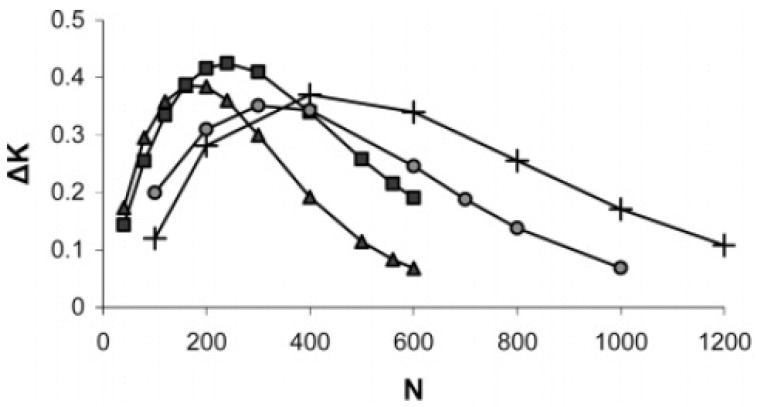
The pH-responsive PLGA grafted nanovalve performance. The change in permeability, ΔK = K_pH=3_ − K_pH=7_, between the open and closed states are plotted as a function of degree of polymerization of the grafted chains for grafting densities 0.05 *a*^−2^ (triangles) and 0.025 *a*^−2^ (squares) at R) 800 Å and 0.05 *a*^−2^ (circles) and 0.025 *a*^−2^ (crosses) at R = 1,600 Å. Reprinted with permission from reference [39]. Copyright (2007) American Chemical Soceity.

To design a smart flow control system, it is necessary to analyze the gating effect as a function of pore size, degree of polymerization, and grafting density. The performance of the nanoporous system at a given grafting condition is determined by the maximum change in permeability between the closed and open states. With reference to equation 1, we characterized the change in solvent permeability by the difference Δ*K* between permeability values at pH = 3 and 7. In Figure 11, ΔK = K_pH=3_ − K_pH=7_ is plotted as a function of the degree of polymerization *N* for two different grafting densities in pores of *R* = 800 and 1,600 Å. At low degrees of polymerization the value of Δ*K* increases with *N*. When the grafted layer thickness in the closed state is comparable to the pore radius, an increase in N continues to decrease *K* in the open state without causing comparable reduction in *K* in the closed state. Consequently, Δ*K* reaches a maximum value and then begins to decrease with increasing *N* as indicated by the results. The *N* value at which Δ*K* reaches a maximum increases with decreasing grafting density and increasing *R*. 

## 4. Conclusions

Smart nanopores based on stimuli-sensitive polymers provide autonomous flow control and allow signal-responsive mass-transport, as well as size- and charge-selective separation of molecules and nanoparticles. Since these smart nanopores can be designed to respond to specific external stimuli including temperature, pH and light, they are very attractive for applications in controlled drug-delivery systems, nanofluidic valves, and nanoporous separation membranes. 

A majority of the work concerning smart flow control based on polymer brushes has been carried out on porous membranes. While relatively less explored, a great potential for smart flow control systems is in the area of on-chip programmable microreactors/analysis systems. A key attraction of the smart nanopore functionalized with grafted polymers is that it can be designed to remain in a given conformational state over a wide range of a stimulus to which it is responsive and then undergo a drastic conformational change when that stimulus is varied over a narrow interval near the critical value. The critical value at which the transition occurs can be tuned to have a specific value, either by inserting different conformers in the polymers or by changing the degree of polymerization. By judiciously designing a series of microreactors, each equipped with its own smart nanovalve and a trigger point, it is possible to fabricate programmable/autonomous on-chip chemical analysis systems. The advancement in nanofabrication capabilities enable the preparation of nanopores with diameters down to a few nanometers, and the next logical step is to fabricate responsive nanofluidic devices by incorporating smart polymer brushes grown by the ATRP method. 

In this work, we presented a model to describe responsive flow control through a smart nanopore. The model is based on the mean-field approach to determine the conformation of the polymer brush layer as a function of solvent quality as well as a continuum approach for calculating the flow through the nanopore. This design allows for a broad range of pore sizes from fully open sub-micrometer pores (colloidal size range) to completely closed pores (molecular filtration through the polymer network). The combination of changes in both the volume and the pore size of the membrane enables not only tuning and switching of the permeability, but also adds an opportunity to regulate a broad range of properties.
